# The problem of suicide among Amerindians in Camopi-Trois Sauts, French Guiana 2008–2015

**DOI:** 10.1186/s12888-018-1670-6

**Published:** 2018-04-11

**Authors:** Rémi Pacot, Basma Garmit, Marianne Pradem, Mathieu Nacher, Paul Brousse

**Affiliations:** 1 Département des centres délocalisés de prévention et de soins, Centre Hospitalier de Cayenne, 97300 Cayenne, French Guiana; 2 Rectorat de la Guyane, 97300 Cayenne, French Guiana; 3 Centre d’Investigation Clinique, INSERM 1424, Centre Hospitalier de Cayenne, 97300 Cayenne, French Guiana

**Keywords:** Suicide, Suicide attempt, Amerindian, French Guiana, Adolescents, Alcohol, Spirit

## Abstract

**Background:**

Suicide within the Amerindian community of Camopi (1741 inhabitants) in French Guiana has been an increasing problem widely reported in the media leading the French Government to mandate a parliamentary mission to investigate the matter. The purpose of the study was to describe this phenomenon and identify factors associated with suicide attempts.

**Methods:**

A retrospective observational study was conducted from the health centers’ medical records. All suicide attempts and suicides committed between 2008 and 2015 by Amerindians living in Camopi and Trois Sauts were compiled. Contextual factors and suicide representations were also analyzed.

**Results:**

During the study period, the annual attempted suicide rate and the suicide rate were higher in the last 3 years. The overall annual rate was equal to 6.9/1741 or 396 per 100, 000 inhabitants for attempted suicide and 172 per 100,000 inhabitants for suicide, which is more than 10 times higher than the suicide rate in mainland France. The mortality rate was 30.4% versus 8.2% in mainland France. The 10–20 year-old age group represented 70% of suicide deaths. There was no significant difference between genders. A recent death and interpersonal conflict were the main stressful life events reported by respondents (55 and 52%, respectively). Alcohol addiction (30% of the respondents) was associated with suicide attempts under the influence of alcohol (*p* = 0.03). Repetition of suicide attempts was associated with cannabis consumption (*p* = 0.03). Depression was reported among 45% of the respondents. A third of respondents reported having been abused during their childhood. Over half of respondents reported that their suicide attempt was motivated by a *spirit* (58%).

**Conclusions:**

Despite limitations due to the small population size and limited time frame, this is the first study to describe the epidemiology of suicide among Amerindians living in Camopi. In contrast with other French territories, the suicide rate was very high, the sex ratio was balanced and younger age groups were most affected.

## Background

Although there are marked variations, suicide kills more persons worldwide than wars or natural disasters [1]. In 2012, the global age-standardized suicide rate was 11.4 per 100,000 inhabitants (15 among men and 8 among women). Among industrialized countries, France has a relatively high suicide rate with about 195,000 suicide attempts and 12,000 suicides [2]. Although completed suicide mostly affects men, suicide attempts mostly affect women in the 15–19-year-old age group, followed by women in the 40–50 -year-old age group. From a geographical point of view, there are differences between French regions, the suicide rate ranging from 9 per 100,000 in Île-de-France to 28 per 100,000 in Bretagne.

The national strategy of suicide prevention is articulated around four priority interventions which are: prevention, reduction of access to lethal means and improvement of care and of epidemiological knowledge. In France, INSERM –CépiDC (French National Medical Research Institute) releases essential indicators on suicide that are based on medical death certificates. However, in French Guiana death certificates are not always performed because of the isolation of certain villages and the cultural barriers in some populations who bury their dead before any investigation.

For 2013, the CépiDC tallied 16 suicides in French Guiana for a total population of 246,507 inhabitants, thus corresponding to a suicide rate of 6.5 per 100,000 inhabitants, a rate that is probably under-estimated. Moreover, it was not possible to disaggregate this data per municipality. In contrast to Mainland France, there is no regional observatory for suicide in French Guiana. The population in French Guiana is very diverse in terms of culture, language, socioeconomic level. Most people live along the coast in one of the three main cities Saint Laurent du Maroni, Kourou, and Cayenne. About 20% of the population, mostly Maroons and Amerindians lives along the Maroni and Oyapock rivers bordering Suriname and Brazil, respectively. In 2006, a study on suicides among Wayana/Emerillon Amerindians living on the French bank of the Maroni river revealed a suicide rate of 280 per 100,000 inhabitants [3]. The study also showed that persons under the age of 25 years, and notably those <15 years, were most affected (70%). Suicide affected both sexes.

In contrast with mainland France, suicide among Wayana Amerindians starts at a very early age and declines thereafter.

On the Oyapock river, which delineates the border with Brazil, the municipality of Camopi-Trois Sauts has also been affected by suicides. Wayãpi and Teko Amerindians have resided there since the 1950’s and the villages have rapidly grown with 1741 persons in 2014 (https://www.insee.fr/fr/statistiques/2011101?geo=DEP-973). Hunting, fishing, gathering and slash and burn agriculture are the traditional means of subsistence. Wayãpi and Teko populations have long remained in isolation, with strict regulations for accessing their territories, notably for the protection against “western” communicable diseases. However, for the past two decades they have been confronted with accelerated modernization, the consequences of welfare benefits, the environmental, health, and social consequences of illegal gold mining. Although there are primary schools in the remote villages, there are no high schools and the children thus need to go to other towns to continue their education. All these changes have led to psychological suffering, notably addictions and suicidal behavior. The main objective was thus to tally the suicide attempts and the suicides between 2008 and 2015. The secondary objectives were to identify risk factors and to grasp some of the representations of suicide in this population.

## Methods

### Study type

The study was observational and retrospective, based on data from all paper medical records from the study period consulted at the health centers by the first author Rémi Pacot. Patient records also included responses from a questionnaire that was systematically administered by Rémi Pacot to persons who had attempted suicide between 2008 and 2015 and observations from the Cayenne hospital mobile psychiatry unit. The aim of this questionnaire was improving care and refining propositions for prevention and post-suicide attempt interventions in the wake of a parliamentary report in 2015 on suicide among Amerindians. Psychiatric diagnoses were made by psychiatrists from the mobile unit but no psychometric tools were used.

Data collection took place during three 1 week periods in June, July and August 2016. The study period lasted from January 1st, 2008 to December 31st, 2015. The unreliability of data before that did not allow us to study earlier periods.

### Study population

The study population consisted of French Amerindians residing in the Camopi-Trois Sauts municipality who committed suicide or attempted suicide during the 2008–2015 study period. Suicide and attempted suicide ascertainment: at the health center two amerindian nursing assistants from the village were precious informants; in addition, medical records were consulted, the information systems of the health centers, of the emergency medical teams based in Cayenne (SAMU which can send a helicopter to transfer patients), and of Cayenne Hospital (in case patients were transferred there without passing through the health center).

### Exclusion criteria

The following were excluded: Brazilian citizens; French of non-Amerindian origin; Accident victims (drowning, boat accident, hunting accident or other for which intention to die was not established); Suicidal events that took place before January first 2008 or after December 31st 2015.

The questionnaire was constructed by the authors for the purpose of the study and administered face to face by Rémi Pacot to ensure homogeneity. Oral information was given to the respondents to explain the purpose of the interview. Their understanding of its aims was verified by Rémi Pacot. Before starting, all persons were informed that they could refuse to answer all or parts of the questionnaire. Questions that were not understood were reformulated until they were understood. Interviews took place in French conducted by Rémi Pacot, or more rarely in Teko or Wayãpi if necessary, with the help of a local health center mediator. The duration of the interview was about 30 min. The questionnaire dealt with general information, the current context, life events in the year before the suicide attempt, vulnerability factors, the use of addictive substances, the place of the health professionals, their conception of suicide and what optimal care would be required for them.

### Analysis

A descriptive analysis was performed. Frequencies and means were compared using Chi2 and Student’s tests. The statistical significance level was 5%.

Because of differences in the categorization of age classes in the demographic data and the categories in the reference population, it was not possible to calculate age-standardized rates. The comparison of suicide rates and suicide attempt rates with mainland France was thus performed using crude rates per 100,000, and rates for a given age group. The comparison may be criticized but it was nevertheless quite striking.

The study concerned anonymized monocentric data (Centres Délocalisés de Prévention et de Soins (CDPS), Department of the remote Health Centers) and was reviewed by the Cayenne Hospital Ethical Committee to ensure that it respected the three pillars of Ethics (well meaning, respect of persons, and justice) and then reported to the national authorities: Commission Nationale Informatique et Libertés (French Data Protection Authority, CNIL N° 1996146).

## Results

### Census of suicides and suicide attempts

**Fig. 1:**
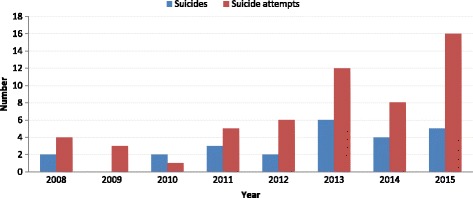
Evolution of the number of suicides and attempted suicides in Camopi-Trois Sauts district

Between 2008 and 2015, 24 suicides were identified at the health centers in Camopi-Trois-Sauts district (annual average 3/1741 or 172 per 100, 000 (95% CI = 36–502 per 100,000)). One person died of unknown cause. During the study period, there was a marked trend towards increased incidence for suicide (linear trend Chi square, *P* = 0.02) (Fig. 1). Thus, for 2013–2015 the average rate for suicide was 287 (95% CI = 93–668) per 100, 000.

## Age

The age of the youngest individual who committed suicide identified by the health center was 13 years. The oldest individual was 41 years old. The median age was 17 years. Overall 17/24 of all deaths were in the 10–20-year-old age group. The suicide method was mostly hanging (18 hangings, five suicides by firearm, one using medication).

## Sex

There were 14 men and 10 women (sex ratio = 1.4). There were no significant differences between men and women (*p* = 0.31).

**Fig. 2:**
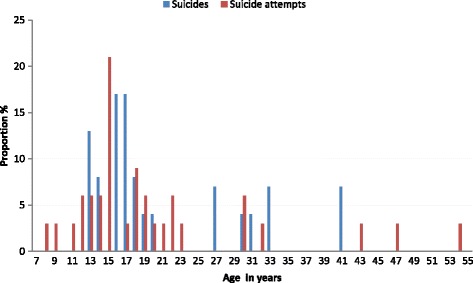
Frequency of suicides by age between 2008 and 2015, in Camopi-Trois Sauts district

Figure 2 shows the frequency of suicides by age between 2008 and 2015, in the Camopi-Trois Sauts district.

### Suicide attempts (SA)

#### Number of suicide attempts

Between 2008 and 2015, there were 55 reported suicide attempts (does not include completed suicides). Figure 1 shows the number of suicide attempts per year. As for suicides, there were more suicide attempts in the 3 last years. Overall the average annual rate was 6.9/1741 or 396 (95% CI = 161–826) per 100, 000 inhabitants for attempted suicide. The temporal trend for suicide attempts showed a significant increase (linear trend Chi square, *P* = 0.001). Thus, for 2013–2015 the average rate for suicide attempts was 689 (95% CI = 356–1200) per 100, 000.

The median age during the study period was 17 years. Figure 2 breaks down the data by age. The sex ratio was 1.06. Of the 55 identified suicide attempts, the method had been specified for 34 patients (One by medication, one using a cutting object, one using a firearm, and the remaining 30 by hanging).

### Lethality

Of all persons attempting suicide, 30.4% died.

### Other aspects

Thirty-three people answered the questionnaire. Nineteen of 33 (57%) respondents lived as a couple and 32/33 (97%) had brothers or sisters; 21/33 respondents (64%) still lived with their parents. On average, there were six people in the household (range: 1–13); 14/33 (42%) had a job or were schooled. Table 1 synthesizes responses regarding the triggering life events, predisposing factors, problems linked to addictive substances, and representations of suicide and spiritual aspects.

**Table 1:** Proximal and contextual factors of suicide acts in Camopi Trois Sauts, French Guiana 2008–2015

	Situation	*N* /33	Percent
Life event	Conflict	17	51
	Separation	4	12
	Problem with the law	3	10
	Unemployment or school interruption	11	33
	Professional or financial problem	16	48
	Mourning	18	55
	Domestic violence	9	27
	Health problem	9	27
Suicide vulnerability factor	Suicide in the family	20	61
	Repeated suicide attempt	8	24
	History of suicide attempt	14	42
	Remembering the date of suicide	2	7
	Depression	15	45
	Violence or impulsivity	6	18
	Childhood abuse	11	33
	Abuse in the household	10	30
	Abuse by kin	8	24
Substance abuse problems	Marijuana consumption	14	42
	Alcohol consumption	25	76
	Suicide attempt + marijuana	1	3
	Suicide attempt + alcohol	11	33
	Dependency	11	33
	Dependencyto alcohol	10	30
	Dependency to cannabis	3	9
Conception of suicide, spirituality	Reincarnation of body	7	21
	Reincarnation of spirit	11	33
	Life after death	8	24
	Voices heard during suicide attempt	12	36
	Voices heard at other moments	7	21
	Suicide attempt motivated by spirit	19	58
	Suicide attempt as a message to close ones	5	15
	Suicide attempt following melancholia	21	64
	Suicide attempt taken care of in the village	8	24
	Religion	5	15

Among the two most cited events, a recent death was reported by 18/33 (55%) respondents, and a notable conflict was reported in 17/33 (52%) respondents. This variable was not significantly linked to gender or household size, or to residing with one’s parents; finally, professional or financial problems were also mentioned in 16/33 (48%) respondents.

When looking at the number of notable life events that occurred the year prior to the suicidal act, 31/33 (94%) reported at least one event, 10/33 (30%) reported less than 3, 11/33 (33%) reported 3 or 4 events and 12/33 (36%) reported between 5 and 8 life events.

### Suicide vulnerability factors

Table 1 shows that 61% of the surveyed persons had previously experienced a suicide or a suicide attempt in their family, either before or after their own suicide attempt (for 45% it was a first degree relative and for 12% a second degree relative). Twenty four percent of the surveyed persons had reattempted suicide at least once, notably those who smoked marijuana.

Depression was frequently reported, notably among those living in a larger household (7 vs. 5, *p* = 0.0014). A third of the surveyed population reported having been abused during their childhood, often in their own household.

Marijuana consumption was frequent and seemed associated with the repetition of suicide attempts (*p* = 0.032); 75% of those who had repeated a suicide attempt smoked marijuana, and 43% of marijuana smokers had reattempted suicide.

Regarding alcohol consumption, 83% of the respondents in Camopi and 60% in Trois-Sauts, consumed non-traditional alcohols such as beer or rhum; 33% of those surveyed recognized having enacted their suicidal wish under the influence of alcohol. The mean age of first non-traditional alcohol consumption was 16 years, (range = 16–20 years).

A total of 11/33 of the surveyed population (33%) declared being dependent from a substance. There was a significant relationship between alcohol dependency and the suicidal act when inebriated (*p* = 0.03).

### Place and role of health professionals

Concerning the relation with healthcare professionals 17/33 (52%) declared having needed professional help at a given moment in order to face the pressure of a suicidal process; 19/33 (58%) of those declaring they had needed help said a professional was available when they needed one; 22/33 (66%) of persons in need declared they spontaneously went to consult the health center; 19/33 (58%) were in favor of establishing a permanent psychiatric team in Camopi to deal with the problem of suicide and 22/33 (66%) for addiction problems. The rest of the population however felt this was inappropriate and useless; 18/33 (55%) had previously consulted Cayenne hospital’s mobile psychiatry unit; finally 28/33 (84%) of those who had consulted the mobile psychiatry unit were satisfied with the intervention.

### Conception of suicide and management in the Amerindian culture

The synthesis of these aspects is indicated in Table 1. The majority of persons did not believe in life after death and did not have any religion. Some referred to spirits at the time of the suicide attempt, or beyond that period.

**Fig. 3:**
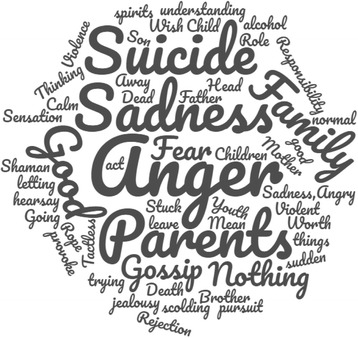
Word cloud representing the most frequent words used by Amerindians having attempted suicide in Camopi Trois Sauts, French Guiana, 2008–2015

An open question, at the end, allowed the respondents to use their own words to describe their feelings about suicide. We did not collect long accounts and some did not wish to respond. A word cloud captured the recurrent themes (Fig. 3).

## Discussion

The problem of suicide in autochthonous populations is not new [4–16], but it is neglected [11]. This is the first epidemiological study on suicide in the population of the Camopi-Trois Sauts district. It shows a gradual increase of suicide events between 2008 and 2015. It is difficult to say whether this trend is stable or whether it reflects an transient phenomenon [17], a Werther effect [18, 19], suggested by the frequency of suicidal events in the close family [20]. The present study had several limitations: case ascertainment, questionnaire development and administration and outcome ascertainment were potential limitations; even though at the scale of a small village suicide was a major problem, the absolute overall number of events per year was “small”, which may lead to wide fluctuations and more extreme rates; some of the data was declarative, which may have biased responses towards more socially desirable answers; and finally the study period may have been too short to witness long term trends. However, despite broad confidence intervals in the annual estimates, even the lower boundary of the 95% confidence interval in Camopi-Trois Sauts was still higher than estimates in France (36 vs. 16 per 100,000, respectively).

Elders in the village reported that, in the past, it was not exceptional for persons to want to end their life, but that it was not as frequent as in recent years [21]. Thus there seemed to be a real increase of suicide. This trend has also been observed in other indigenous populations. The observed suicide rate observed in Camopi-Trois Sauts is close to what was reported on the upper Maroni in 2006 (280 per 100,000) [3] with similar characteristics in terms of age and gender. Although age categorization differences made it impossible to standardize suicide rates by age, the crude comparison with mainland France is still quite striking: 172/100000 versus 16/100000 (2015), hence over 10 times more [22, 23]. In practice, this means that for the 2008–2015 period 1.4% (95% CI = 0.9–2.1) of the population of Camopi-Trois Sauts committed suicide, more specifically 3% of the 15–29 year-old age group; it also means that 3.2% (95% CI = 2.4–4.2) of the same population, and 6.5% (1 in 15 persons) of the 15–29 year-old age group, had attempted suicide. The small size of the villages implies that nearly everyone has seen or felt the impact of this trauma. The epidemiologic studies of different autochthonous populations usually observe high suicide rates, with a similar epidemiological profile in terms of age but they often report a male predominance, which was not the case in Camopi-Trois Sauts. Despite similarities, the incidence observed in French Guiana seems particularly high relative to these studies (51.7/100000 in young men in Alaska [16, 24, 25]). It is possible that this very high rate is a peak value measured on a small sample at the apex of a transient phenomenon, which may subsequently drop to pre-episode levels. However, it underscores the considerable intensity of the phenomenon.

Apart from the sole quantitative aspect, there were also specific differences with mainland France, with a very young age at the time of the suicidal act in Camopi-Trois Sauts (71% were less than 25 years) whereas, in France the incidence of suicide was 6.4/100000 for the 15–24 year-old age group and 40.3/100000 for the 85–94 year-old age group.

The average yearly suicide attempt rate was 396 per 100,000 inhabitants for Camopi-Trois Sauts, a rate over twice greater than the crude rate observed in mainland France (177 per 100,000 inhabitants).

In addition, the lethality rate of 30.4%, which is about four times higher than in mainland France (8.2%), shows the radical nature of suicidal acts in Camopi-Trois Sauts, where most suicide attempts were by hanging.

The principal life events preceding suicide were the death of someone close, interpersonal conflicts, professional or financial problems, an interruption of school or unemployment, and finally health problems. This is relatively similar to the literature. It is of note that persons often cumulated several difficult life events, and nearly all reported at least one.

It is not easy to intervene on the occurrence of these first life events. However, the prevention and support provided aim at reducing suicidal events.

Concerning the prevention and management of interpersonal conflicts, traditional leaders and traditional modes of regulation must be rehabilitated [26] in partnership with the municipality (https://www.interieur.gouv.fr/SG-CIPDR/Outils-et-initiatives/Les-outils-du-Maire/Le-guide-d-installation-du-CDDF). The chronic problem of illegal gold mining, which is very real in these remote areas, adds stress and division in the community. The acknowledgement of psychological suffering and mental health and addiction issues by psychiatry professionals assisted by local mediators seems important [5, 6, 27, 28]. Childhood abuse is a known risk factor for suicide [29], it was reported in a non-negligible number of cases. The creation of the regional cell for the well-being of populations of the interior (CeRMEPI) has an important coordinating role for actions that aim at preventing the life events triggering suicide.

The frequency of conflicts, the fear of gossip, and the resonance of life events may be linked to the small size of the villages and the geographic and family proximity of suicides and suicide attempts. Thus 61% of those surveyed revealed a suicidal event of a family member, 45% of whom were first degree relatives. These percentages are the proof a serial phenomenon that must be contained.

As elsewhere suicide was closely associated with alcohol [30, 31]. Most persons who were drunk during their suicide attempt were addicted to alcohol. The repetition of a suicide attempt was significantly linked to the consumption of marijuana, a potentially interesting information for prevention [27]. Recent studies of a different design seemed to suggest that alcohol and marijuana consumption were greater than in the general population [32, 33]. Psychiatric risk factors have a clear influence on suicidal acts. Untreated depression is considered as the first cause of suicide [27, 28].

The unusually high lethality rate could raise the question of ascertainment bias and the underestimation of suicide attempts relative to suicides. However, given the size of the villages, underestimation seemed less likely than at the scale of larger territories. Therefore, the high lethality rate, and the lethal methods used in suicide attempts seem more indicative of the acuity of lethal intent in attempted suicides. The balanced sex ratio, which is unusual, could suggest particularities regarding gender relations with particular psychological distress among young women. We did not have any data to further explore this hypothesis but previous studies showed 11% of women experiencing forced first intercourse [33]. The absence of secondary schools, leads to separation between youths and their family as they move to other towns to continue studying. This presumably leads them to grow apart from traditional culture and fuels intergenerational conflicts, situations that may exacerbate psychological suffering when they come back to their village, isolated, torn between traditional and modern cultures in a context where suicide is omnipresent [34]. The regional cell for the well-being of populations of the interior (CeRMEPI), trained local mediators and educators to assist those with mental health problems, and prevent suicide, rapid response psychiatric teams may have an important role to relieve psychological suffering among youths in these areas. The above interventions (CeRMEPI, on site schools, trained educators and mediators, psychiatric teams to implement an emergency medico-psychologic response in less than 24 h) may help alleviate psychological suffering and suicidality. The interviewed persons were favorable to the idea of psychiatric help. However, these interventions should be prospectively evaluated and the present efforts to quantify the problem of suicide should be pursued in order to see if there is any progress following the interventions. A recent parliamentary mission published a report on the subject of suicide in this area [35]. The report detailed 37 propositions to improve well being and reduce suicidality in these territories, notably the presence of a permanent psychiatric facility in Camopi, and emergency medico-psychological task force able to respond within 24 h, and the creation of an observatory of suicide, which definitely would be useful to follow future trends.

## Conclusion

Despite limitations linked to case and outcome ascertainment, small population size and limited time frame, the study of the data from the health centers allowed quantifying and characterizing the phenomenon of suicide in Camopi-Trois Sauts. There was a high suicide rate with an increasing trend, (1 person out of 15 in the 15–29 year-old age group had attempted suicide). Suicides essentially concerned young persons of both sexes, most occurring between 10 and 20 years old. These suicides seemed to follow one or more precipitating factors, notably interpersonal conflicts, often in a context of alcohol addiction and isolation. The consumption of marijuana seemed associated with recurrent suicidal acts. Persons who had attempted suicide were willing to accept psychiatric help or addiction treatment. The suspected determinants were linked to the individual (mental illness), to social aspects (absence of high schools) and to culture (conflict management) [36]. Thus suicide prevention will require the therapeutic alliance between the community, traditional healers [5], health professionals, and educators.

## Abbreviations

CDPS: Centres Délocalisés de Prévention et de Soins
CeRMEPI: Cellule Régionale pour le mieux être des populations de l’intérieur
CNIL: Commission Nationale Informatique et Libertés
INSERM: Institut National de la Santé et de la Recherche Médicale
